# Hepatic arterial infusion chemotherapy plus targeted therapy and immunotherapy versus systemic chemotherapy for advanced intrahepatic cholangiocarcinoma: a retrospective cohort study

**DOI:** 10.1097/JS9.0000000000002013

**Published:** 2024-08-07

**Authors:** Zhikai Zheng, Jiongliang Wang, Tianqing Wu, Minrui He, Yangxun Pan, Juncheng Wang, Jinbin Chen, Dandan Hu, Li Xu, Yaojun Zhang, Minshan Chen, Zhongguo Zhou

**Affiliations:** a Department of Liver Surgery, Sun Yat-sen University Cancer Center; b State Key Laboratory of Oncology in South China, Guangdong Provincial Clinical Research Center for Cancer, Sun Yat-sen University Cancer Center, Guangzhou, People’s Republic of China

**Keywords:** advanced intrahepatic cholangiocarcinoma, hepatic arterial infusion chemotherapy, immunotherapy, systemic chemotherapy, targeted therapy

## Abstract

**Background:**

To improve the prognosis of advanced intrahepatic cholangiocarcinoma (iCCA), the authors retrospectively compared the effect and safety of combined hepatic arterial infusion chemotherapy (HAIC), targeted therapy, and immunotherapy with systemic chemotherapy (SC) in unresectable iCCA patients.

**Methods:**

The authors retrospectively enrolled 202 advanced iCCA patients treated with SC or targeted therapy, immunotherapy, and FOLFOX-HAIC combined between March 2015 and June 2023 at our institution. Two hundred two patients were divided into two groups based on the therapeutic regimens. Baseline characteristics and prognosis were reviewed and analyzed.

**Results:**

After 1-to-1 propensity score matching, 76 patients were included in each group. The triple combination therapy group demonstrated longer median overall survival (OS, 20.77 vs. 14.83 months, *P*=0.047), progression-free survival (PFS, 9.07 vs. 6.23 months, *P*<0.001), intrahepatic PFS (11.03 vs. 6.73 months, *P*<0.001), extrahepatic PFS (11.37 vs. 7.13 months, *P*=0.0064), and a higher objective response rate (35.5% vs. 14.5%, *P*=0.003) than the SC group. Fever, thrombocytopenia, elevated ALT, elevated AST, hypoalbuminemia, and hyperbilirubinemia were more common adverse events (AEs) in the triple combination therapy group, while fatigue and anemia were more prevalent in the SC group (*P*<0.05). For grades 3-4 AEs, the rates of elevated ALT were higher in the triple combination group (*P*=0.028).

**Conclusions:**

Compared with SC, triple combination therapy comprising HAIC, targeted therapy and immunotherapy appears to be an effective and safe treatment for advanced iCCA.

## Introduction

Highlights

- Triple combination therapy of hepatic arterial infusion chemotherapy (HAIC), targeted therapy, and immunotherapy was optional for patients with advanced iCCA.
- Triple combination therapy resulted in better survival outcomes, which is superior to systemic chemotherapy (SC).
- The treatment-related adverse events of triple combination therapy were manageable.

Intrahepatic cholangiocarcinoma (iCCA) is an epithelial cell malignancy arising from the biliary tree and is located proximally to the second-degree bile ducts within the liver parenchyma, which is the most common type of hepatic malignancy after hepatocellular carcinoma (HCC)^1^.

Surgery is the only potentially curative treatment for iCCA. However, fewer than 30% of patients are eligible for surgical resection. Most patients present with advanced-stage or metastatic disease at the time of diagnosis^2^. For unresectable iCCA, systemic chemotherapy (SC) with the combination of gemcitabine and platinum is the mainstay of treatment. Nevertheless, the therapeutic effect remains unsatisfactory, with a median overall survival (OS) of less than 1 year^3^. Therefore, various other therapeutic regimens, such as immunotherapy, targeted therapy, and locoregional therapies are used to improve outcomes. Locoregional therapies include transcatheter arterial chemoembolization (TACE), hepatic arterial infusion chemotherapy (HAIC), ablation, and external beam radiotherapy (EBRT)^2^.

Combinational therapy may be more effective and lead to a more durable response, which has been advocated in guideline^4^, and the triple combination therapy of FOLFOX-HAIC, targeted therapy, and immunotherapy has shown potential synergistic effect and promising efficacy results in advanced iCCA^5^. Hence, we conducted this retrospective study to compare the effect and safety of FOLFOX-HAIC, targeted therapy, and immunotherapy with SC for advanced iCCA.

## Materials and methods

### Patients

This was a retrospective cohort study of patients with advanced iCCA who were treated with either SC or the triple combination therapy (FOLFOX-HAIC, TKIs, and anti-PD-L1/PD-1) from March 2015 to June 2023 at our institution. Patients were eligible if they were age 18 years or older; had received a pathological diagnosis of nonresectable or metastatic cholangiocarcinoma; had been treated with either SC or the triple combination therapy; and had an Eastern Cooperative Oncology Group performance status (ECOG PS) of 0–2. Patients meeting the following criteria were excluded: no pathology results; diagnosed with pCCA, dCCA, or combined hepatocellular-cholangiocarcinoma; presented with other malignant tumors or serious medical diseases; and with incomplete follow-up data.

This study was approved by the ethics committee of our institution (Protocol code: B2018-118-01, date: December 2018) and was registered in the Chinese Clinical Trials Registry.

### Procedures

In the triple combination therapy group, patients received FOLFOX-HAIC, targeted therapy (TKIs) plus immunotherapy (anti-PD-L1/PD-1). FOLFOX-HAIC was administered as follows: a catheter/microcatheter was placed in the main feeding hepatic artery, and then the FOLFOX was delivered into the liver in order via a HAIC pump: oxaliplatin, 85 or 130 mg/m^2^, for 2–3 h on day 1; leucovorin, 400 mg/m^2^, for 1–2 h on day 1; fluorouracil, 400 mg/m^2^ in bolus, and then 2400 mg/m^2^ continuously infusion for 23 or 46 h on days 1 and 2^6^. HAIC was performed every 3 weeks. Immunotherapy was performed 0–1 day prior to HAIC and administered for each HAIC cycle. Target therapy was administered 0–1 week prior to the initial HAIC, and was not discontinued before and after each HAIC cycle.

In the SC group, patients received intravenous chemotherapy with the first-line GemCis regimen or GemOx regimen. In the GemCis group, patients received gemcitabine (1000 mg/m²) and cisplatin (25 mg/m²) on days 1 and 8 every cycle, which were administered every 3 weeks. In the GemOx group, patients accepted oxaliplatin (85 mg/m^2^) on day 1, and gemcitabine (1000 g/m^2^) on days 1 and 8 every cycle per 3 weeks. During the treatment of SC, patients might be combined with anti-PD-L1/PD-1 or TKIs after a multidisciplinary review.

### Baseline characteristics, outcomes, and follow-up

Baseline characteristics before treatment were collected and analyzed. Tumor response was evaluated based on the Response Evaluation Criteria in Solid Tumors (RECIST) 1.1. The primary outcome was overall survival (OS), defined as the time from first treatment to death regardless of any cause. The secondary outcomes were progression-free survival (PFS), defined as the time from the first treatment until progression, or death from any cause, or the last follow-up date; intrahepatic PFS (IPFS), defined as the time from the first treatment until intrahepatic tumor progression, or death from any cause, or the last follow-up date, no matter the extrahepatic metastasis; extrahepatic PFS (EPFS), defined as the time from the first treatment until extrahepatic tumor progression, or death from any cause, or the last follow-up date, no matter the intrahepatic metastasis; objective response rate (ORR), defined as the proportion of patients with complete response (CR) or partial response (PR); disease control rate (DCR), defined as the proportion of patients with ORR plus stable disease (SD); and adverse events (AEs) according to the National Cancer Institute Common Terminology Criteria for Adverse Events (NCICTCAE) 5.0.

### Statistical analysis

Categorical variables were described as frequencies and percentages. Continuous variables were described as the mean*±*SD or median*±*interquartile range according to parametric and nonparametric variables. Categorical variables were compared by *χ* ^2^ tests or Fisher’s exact tests, while continuous variables were compared by Student’s *t*-tests or rank sum tests. To minimize the effect of selection bias and potential confounding between the two groups, we performed 1-to-1 propensity score matching (PSM) considering the baseline variables. PSM was conducted by the R package ‘MatchIt’, and the caliper width was 0.2. Kaplan–Meier curves were generated to estimate OS, PFS, IPFS, and EPFS, and differences between curves were evaluated using a log-rank test. All statistical analyses above were completed by SPSS 26.0 and R 4.3.2. Two-tailed *P-*values <0.05 were considered statistically significant.

## Results

### Baseline characteristics of the patients

Between March 2015 and June 2023, a total of 202 patients who met the criteria were included in this study: 107 patients were treated with SC, and 95 patients were treated with the triple combination therapy. The baseline characteristics of the two groups were summarized in Table 1. In the full cohort, there were more patients with lymph node or distant metastasis (94.4 vs. 81.1%, *P*=0.003), and TNM stage III–IV (95.3 vs. 84.2%, *P*=0.008) in the SC group, and more patients with MVI (56.8 vs. 37.4%, *P*=0.006) in the triple combination group. After 1-to-1 matching, there were 76 patients in both groups and no significant difference between the two groups (*P*>0.05).

**Table 1:** Baseline characteristics of patients before propensity score matching (*n*=202) and after propensity score matching (*n*=152).

	Before PSM			After PSM		
Variable	Systemic chemotherapy (*n*=107)	Triple combination therapy (*n*=95)	*P*	Systemic chemotherapy (*n*=76)	Triple combination therapy (*n*=76)	*P*
Age (>60/≤60)	37/70 (34.6%/65.4%)	36/59 (37.9%/62.1%)	0.624	28/48 (36.8%/63.2%)	27/49 (35.5%/64.5%)	0.866
Sex (men/women)	69/38 (64.5%/35.5%)	60/35 (63.2%/36.8%)	0.845	49/27 (64.5%/35.5%)	48/28 (63.2%/36.8%)	0.866
Hypertension (yes/no)	17/90 (15.9%/84.1%)	18/77 (18.9%/81.1%)	0.566	14/62 (18.4%/81.6%)	13/63 (17.1%/82.9%)	0.832
Diabetes (yes/no)	4/103 (3.7%/96.3%)	8/87 (8.4%/91.6%)	0.160	4/72 (5.3%/94.7%)	4/72 (5.3%/94.7%)	1.000
WBC (×10^9/l)	7.51±2.82 [3.58–20.40]	7.35±3.53 [2.88–15.91]	0.921	7.52±2.73 [3.58–20.40]	7.15±4.35 [2.88–15.91]	0.905
Neutrophils (×10^9/l)	5.16±2.80 [1.63–18.50]	5.11±2.94 [1.72–13.33]	0.947	5.20±2.54 [1.63–18.50]	5.04±3.40 [1.72–13.33]	0.966
HGB (g/l)	131±20 [79–173]	133±24 [87–182]	0.500	131±14 [85–167]	130±19 [87–168]	0.646
PLT (×10^9/l)	229±110 [99–586]	221±123 [64–634]	0.383	226±95 [99–539]	216±124 [64–634]	0.452
ALT (U/l)	26.4±25.4 [6.6–258.3]	25.1±18.7 [6.6–127.8]	0.947	26.5±20.5 [7.0–135.9]	25.5±19.4 [6.6–102.1]	0.919
AST (U/l)	31.7±19.7 [10.9–444.7]	29.2±23.1 [12.4–128.4]	0.406	31.9±16.5 [10.9–164.3]	29.5±23.5 [12.4–128.4]	0.480
ALB (g/l)	42.5±5.7 [28.5–50.6]	42.8±6.5 [27.2–50.1]	0.817	42.6±5.7 [30.6–50.6]	43.0±6.7 [27.2–50.1]	0.911
TBIL (μmol/l)	11.9±8.5 [4.1–239.3]	11.9±9.3 [4.5–82.3]	0.925	11.8±7.8 [4.1–51.8]	11.7±9.5 [4.5–82.3]	0.360
Creatine (μmol/l)	63.4±22.7 [33.8–141.5]	66.0±22.5 [40.2–110.6]	0.903	62.2±22.5 [40.2–141.5]	65.4±21.5 [40.2–110.6]	0.712
PT (s)	11.6±0.9 [9.8–18.2]	11.6±1.3 [9.6–14.9]	0.820	11.6±1.0 [9.9–16.0]	11.8±1.3 [9.6–14.9]	0.922
AFP, ng/ml (≥25/<25)	9/98 (8.4%/91.6%)	16/79 (16.8%/83.2%)	0.069	9/67 (11.8%/88.2%)	10/66 (13.2%/86.8%)	0.806
CA19–9, U/ml (≥35/<35)	64/43 (59.8%/40.2%)	57/38 (60.0%/40.0%)	0.978	46/30 (60.5%/39.5%)	48/28 (63.2%/36.8%)	0.738
ALBI (3/2/1)	1/26/80 (0.9%/24.3%/74.8%)	0/24/71 (0/25.3%/74.7%)	1.000	0/16/60 (0/21.1%/78.9%)	0/20/56 (0/26.3%/73.7%)	0.445
Number of tumors (multiple/single)	63/44 (58.9%/41.1%)	65/30 (68.4%/31.6%)	0.160	52/24 (68.4%/31.6%)	55/21 (72.1%/27.6%)	0.594
Max diameter of tumors, mm (≥100/<100)	20/87 (18.7%/81.3%)	21/74 (22.1%/77.9%)	0.547	19/57 (25.0%/75.0%)	16/60 (21.1%/78.9%)	0.563
Macrovascular invasion (yes/no)	40/67 (37.4%/62.6%)	54/41 (56.8%/43.2%)	0.006	37/39 (48.7%/51.3%)	38/38 (50.0%/50.0%)	0.871
Lymph node/distant metastasis (yes/no)	101/6 (94.4%/5.6%)	77/18 (81.1%/18.9%)	0.003	70/6 (92.1%/7.9%)	70/6 (92.1%/7.9%)	1.000
Differentiation (moderately/poorly)	38/69 (35.5%/64.5%）	26/69 (27.4%/72.6%）	0.214	22/54 (28.9%/71.1%)	20/56 (26.3%/73.7%)	0.717
TNM stage (III–IV/II)	102/5 (95.3%/4.7%)	80/15 (84.2%/15.8%)	0.008	71/5 (93.4%/6.6%)	71/5 (93.4%/6.6%)	1.000
Cycles of SC/HAIC	3±4 [1–9]	4±3 [1–8]	0.762	3±4 [1–9]	4±3 [1–8]	0.790

AFP, alpha fetoprotein; ALB, albumin; ALBI, albumin to bilirubin score; ALT, alanine transaminase; AST, aspartate transaminase; CA19–9, carbohydrate antigen 19–9; HAIC, hepatic arterial infusion chemotherapy; HGB, hemoglobin; PLT, platelet; PSM, propensity score matching; PT, prothrombin time; SC, systemic chemotherapy; TBIL, total bilirubin; TNM, tumor–node–metastasis; WBC, white blood cell.

### Tumor response and patients’ survival outcomes

**Figure 1:**
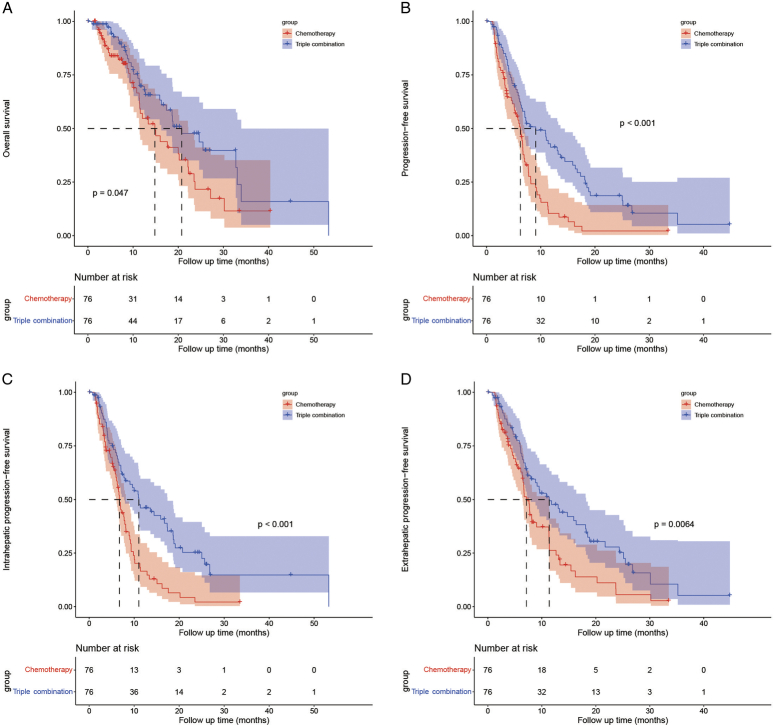
Kaplan–Meier curves of overall survival (A), progression-free survival (B), intrahepatic progression-free survival (C), and extrahepatic progression-free survival (D) between the two groups after matching.

In the matched cohort, the triple combination therapy group had longer median OS: 20.77 (95% CI: 13.60–27.93) months vs. 14.83 (95% CI: 9.98–19.69) months, *P*=0.047, longer median PFS: 9.07 (95% CI: 4.72–13.41) months vs. 6.23 (95% CI: 5.17–7.29) months, *P*<0.001, longer median IPFS: 11.03 (95% CI: 6.77–15.30) months vs. 6.73 (95% CI: 5.91–7.56) months, *P*<0.001, and longer median EPFS: 11.37 (95% CI: 7.25–15.48) months vs. 7.13 (95% CI: 6.00–8.27) months, *P*=0.0064 (Fig. 1) than the SC group. Besides, the ORR was higher in the triple combination therapy group than that in the SC group (35.5 vs. 14.5%, *P*=0.003), though there was no statistical difference in DCR (77.6 vs. 63.2%, *P*=0.051).

### Adverse events

Safety was evaluated by assessing AEs in different groups according to CTACE (Table 2). After matching, the rates of all AEs and severe AEs (grades 3–4) showed no significant difference in the two groups (98.7% vs. 94.7%, *P*=0.367; 40.8% vs. 44.7%, *P*=0.623). The triple combination therapy group had higher rates of fever (47.4% vs. 23.7%, *P*=0.002), thrombocytopenia (61.8% vs. 35.5%, *P*=0.001), elevated ALT (69.7% vs. 32.9%, *P*<0.001), elevated AST (89.5% vs. 39.5%, *P*<0.001), hypoalbuminemia (89.5% vs. 53.9%, *P*<0.001), hyperbilirubinemia (55.3% vs. 17.1%, *P*<0.001), and the SC group had higher rates of fatigue (32.9% vs. 10.5%, *P*=0.001) and anemia (77.6% vs. 59.2%, *P*=0.015). Meanwhile, the rates of grades 3–4 elevated ALT were higher in the triple combination group (11.8% vs. 2.6%, *P*=0.028). All patients recovered after supportive treatment and no treatment-related deaths were observed in the two groups.

**Table 2:** Adverse events of patients after propensity score matching (*n*=152).

	Systemic chemotherapy (*n*=76)		Triple combination therapy (*n*=76)		*P*	*P*
Adverse events	Any grade (%)	Grades 3–4 (%)	Any grade (%)	Grades 3–4 (%)	Any grade	Grades 3–4
Fever	18 (23.7%)	2 (2.6%)	36 (47.4%)	4 (5.3%)	0.002	0.681
Abdominal pain	28 (36.8%)	1 (1.3%)	34 (44.7%)	6 (7.9%)	0.322	0.116
Nausea	23 (30.3%)	5 (6.6%)	24 (31.6%)	0	0.861	0.058
Vomit	14 (18.4%)	5 (6.6%)	20 (26.3%)	1 (1.3%)	0.243	0.209
Fatigue	25 (32.9%)	0	8 (10.5%)	0	0.001	—
Rash	9 (11.8%)	0	6 (7.9%)	0	0.415	—
Abdominal distension	15 (19.7%)	0	15 (19.7%)	0	1.000	—
Diarrhea	6 (7.9%)	0	5 (6.6%)	0	0.754	—
Constipation	10 (13.2%)	0	4 (5.3%)	0	0.092	—
Leukopenia	32 (42.1%)	8 (10.5%)	30 (39.5%)	2 (2.6%)	0.869	0.050
Neutropenia	31 (40.8%)	11 (14.5%)	35 (46.1%)	11 (14.5%)	0.513	1.000
Anemia	59 (77.6%)	11 (14.5%)	45 (59.2%)	4 (5.3%)	0.015	0.057
Thrombocytopenia	27 (35.5%)	12 (15.8%)	47 (61.8%)	9 (11.8%)	0.001	0.481
Elevated ALT	25 (32.9%)	2 (2.6%)	53 (69.7%)	9 (11.8%)	<0.001	0.028
Elevated AST	30 (39.5%)	6 (7.9%)	68 (89.5%)	11 (14.5%)	<0.001	0.198
Hypoalbuminemia	41 (53.9%)	0	68 (89.5%)	0	<0.001	—
Hyperbilirubinemia	13 (17.1%)	5 (6.6%)	42 (55.3%)	5 (6.6%)	<0.001	1.000
Elevated creatine	15 (19.7%)	0	16 (21.1%)	1 (1.3%)	0.840	1.000
Total	72 (94.7%)	34 (44.7%)	75 (98.7%)	31 (40.8%)	0.367	0.623

ALT, alanine transaminase; AST, aspartate transaminase.

## Discussion

In the present study, we found that the triple combination therapy was associated with better OS, PFS, IPFS, EPFS, and a higher ORR. Meanwhile, the AEs of the triple combination therapy were manageable.

ICCA is a rare but highly lethal neoplasm that is often diagnosed at an advanced-stage. For patients with advanced iCCA, multiple options should be provided to control tumor growth and improve the quality of life. SC with GemCis is the first-line treatment for patients with unresectable iCCA^7^. Valle *et al*.^8^ revealed that the median OS of patients was 11.7 months (95% CI: 9.5–14.3 months) in the GemCis group. Other regimens, such as GemOx, with a similar median OS and more favorable toxicity profile, are also recommended for iCCA patients, especially in Asia^9,10^. Regardless of the type of chemotherapy administered, the median OS of chemotherapy for all patients with iCCA was 12.6 months (95% CI: 8.7–15.2 months), indicating limited effects^11^.

Considering iCCA is mostly confined to the liver and most patients with iCCA die from intrahepatic tumor progression with biliary obstruction and liver functional failure, locoregional treatment for iCCA is particularly important^12^. HAIC is one such locoregional therapy that could deliver chemotherapy drugs into the hepatic arterial supply of the tumor, leading to a markedly higher concentration in the tumor than in normal parenchyma, thereby improving the antitumor effect^6^. In this study, the median IPFS in the triple combination therapy group was better than that in the SC group. The combination therapy group had better intrahepatic lesion control, due to the high concentration of intrahepatic chemotherapy with HAIC.

In addition, immunotherapy and targeted therapy, as novel treatment approaches, also might be a breakthrough in improving the efficacy of advanced iCCA. In iCCA patients, PD-L1 expression is upregulated in tumor tissue, potentially leading to immune escape and serving as a biomarker of the response to anti-PD-1/PD-L1 immunotherapy^13^. At the same time, iCCA tumor tissue has many other potential targets, such as mutation of IDH, FGFR, NTRK, and HER2^14^. Targeted therapy plus immunotherapy has been reported to increase response rates^15^.

As the extensive interactions and crosstalk between the various treatments in iCCA may enhance treatment efficacy, and the combination of targeted therapy, immunotherapy, and FOLFOX-HAIC has shown a synergistic effect and promising efficacy results in advanced HCC^16^, the triple combination therapy may have more potential and lead to a more durable response in advanced iCCA patients. Consistent with our hypothesis, in the present study, the triple combination therapy was associated with better OS, PFS, IPFS, and higher ORR. Notably, the median EPFS in the triple combination therapy group was better than that in the SC group, demonstrating that targeted therapy and immunotherapy could help control metastatic lesions.

Safety and the incidence of AEs are also important indicators for evaluating the regimen apart from the effect. In our study, the rates of all AEs and severe AEs (grades 3-4) in the two groups were similar. Due to the high concentration of chemotherapeutic drugs in the liver, patients in the triple combination therapy presented more AEs related to liver dysfunction, including elevated ALT, elevated AST, hypoalbuminemia, and hyperbilirubinemia. Conversely, patients in the SC group had higher rates of fatigue and anemia. For severe AEs, the rates of elevated ALT were higher in the triple combination group, but all patients recovered after supportive treatment, and no treatment-related deaths were observed in the triple combination therapy groups. The treatment-related AEs of triple combination therapy were manageable.

There are several limitations in this study. First, this is a single-center retrospective cohort study, which may have a risk of selection bias even after PSM. A randomized controlled trial is ready to set up, which may provide more evidence later. Second, the sample size in our study was not large, which may affect the generalizability of this work. Finally, biological mechanisms to support this phenomenon are not investigated. Further studies are needed.

## Conclusions

In conclusion, our study indicated that, compared with SC, triple combination therapy of FOLFOX-HAIC, targeted therapy (TKIs) and immunotherapy (anti-PD-L1/PD-1) might be an effective and safe treatment for patients with advanced iCCA.

## Ethical approval

This study was approved by the ethics committee of Sun Yat-sen University Cancer Center (Protocol code: B2018-118-01, date: December 2018).

## Consent

Written informed consent was obtained before treatment.

## Source of funding

This work was supported by the Sun Yat-sen University Cancer Center physician-scientist funding (No. 16zxqk04), Wu Jieping Medical Foundation-special fund for tumor immunity (320.6705.2021-02-76), Bethune Fund-Advanced solid tumor project (STLKY2-041), Guangdong Basic and Applied Basic Research Foundation (2022A1515110961), Guangzhou Science and Technology Plan Project (2023A04J2125), National Natural Science Foundation of China (82303893), and Natural Science Fund of Guangdong Province (No. 2024A1515012966).

## Author contribution

Z.Z.: concept and design; Z.Z., J.W., T.W., M.H., Y.P., J.W., J.C., and D.H.: data collection; Z.Z.: data analysis and interpretation; Z.Z., J.W., T.W., and M.H.: drafting the article. All authors contributed to the critical revision of the article and approved the final version to be published.

## Conflicts of interest disclosure

The authors have no conflicts of interest to declare.

## Research registration unique identifying number (UIN)

1. Name of the registry: Chinese Clinical Trial Registry chictr.org.cn.
2. Unique identifying number or registration ID: ChiCTR1800020427.
3. Hyperlink to your specific registration (must be publicly accessible and will be checked): https://www.chictr.org.cn/showproj.html?proj=34369.

## Guarantor

Zhongguo Zhou.

## Data availability statement

The data that support the findings of this study are available from the corresponding author upon reasonable request.

## Provenance and peer review

Not commissioned, externally peer-reviewed.
